# Fast and Furious: Fast Neutron Therapy in Cancer Treatment

**DOI:** 10.14338/IJPT-22-00017

**Published:** 2022-08-05

**Authors:** Konstantin Gordon, Igor Gulidov, Timur Fatkhudinov, Sergey Koryakin, Andrey Kaprin

**Affiliations:** 1 Federal State Autonomous Educational Institution of Higher Education “People's Friendship University of Russia,” Medical Institution, Moscow, Russia; 2 A. Tsyb Medical Radiological Research Center—branch of the National Medical Research Radiological Center of the Ministry of Health of the Russian Federation, Obninsk, Russia

**Keywords:** fast neutrons, cancer treatment, radiation therapy

## Abstract

Fast neutron therapy has been used for decades. In conjunction with recent advances in photonic techniques, fast neutrons are no longer of much oncologic interest, which is not unequivocally positive, given their undoubted therapeutic value. This mini-review recalls the history of medical research on fast neutrons, considers their physical and radiobiological properties alongside their benefits for cancer treatment, and discusses their place in modern radiation oncology.

## Introduction

Over the past 2 decades marked by the emergence of intensity-modulated radiation therapy (RT), volumetric-modulated arc therapy, and image guidance, photon therapy more or less has reached its limits. Its further development is restricted by the physical nature of photons. In this context, hadron-based therapies come to the fore as a novel promising option, with proton therapy and carbon-ion therapy centers being the main focus. By 2023, the number of particle facilities in Europe will be increased to 45 [1]. At the same time, the huge potential of particle therapy remains largely underestimated. For instance, classical fast neutron therapy (FNT), a plausible treatment option for cancers at high risk of local recurrence and radioresistant tumors, has been left behind [2, 3].

Neutrons, one of the first known hadrons, have been applied in medicine since their discovery by Chadwick in 1932 [4, 5]. Despite some remarkable clinical outcomes achieved at the dawn of FNT in the 1930s, the growing evidence of severe complications affecting normal tissues put the clinical use of neutrons on a long pause. The approach was reborn in the 1970s after extensive radiobiological research on the dependence of biological endpoints on the linear energy transfer (LET) of the radiation modality. However, after the great splash of interest in the 1970s and 1980s, by the beginning of the 21st-century neutron facilities have all but disappeared and only a few such centers are still functioning.

The unique radiobiological properties of neutrons allow overcoming the multifaceted mechanisms of resistance executed by tumor cells [6–12]. Regardless of the wide availability of conventional photon radiation units and the growing number of proton and C-ion centers, FNT remains a relevant option for certain tumors [13].

The current number of patients having received FNT is just over 35 000, and the sources are restricted to a few databases. The aim of this review was to recall the history and radiobiology of FNT to discuss the experience of its clinical use in cancer patients, the current state of the field, and its prospects. Numerous important studies illustrating the global FNT experience are listed in Tables 1 and 2.

**Table 1.** Studies evaluating fast neutron therapy for brain, head, and neck tumor locations.

**Reference**	**Publication date**	**N of neutron pts**	**Type of FNT**	**Compared with photons**	**Pathology**	**Outcomes**	**Toxicity**
Caterall [14]	1977	97	Pure	Yes	H&N c-r	1-y OS 53%; 2-y OS 28%	4 pts with G4
Maor [15]	1983	54	Mixed	Yes	H&N c-r	LC 44%; OS 20%	4 pts with G3–4
Errington [16]	1986	43	Pure	No	Sinonasal c-r	5-y LC 50%; 5-y OS 30%	30% complication rate at 27 mo
Mardynsky [17]	1991	27	Mixed	Yes	Laryngeal c-r	2-y OS 81%	3 pts with perichondritis
Liao [18]	2014	14	Pure	No	H&N mucosal melanoma	5-y LC 66%; 5-y OS 21%	4 pts with G3 mucositis, 2 events of osteoradionecrosis
Bucholz [19]	1992	53	Pure	No	Salivary gland ACC	5-y LRC 77%; 5-y OS 33%	19% of G3, 2 events of death
Laramore [20]	1993	13	Pure	Yes	Salivary gland ACC	10-y LC 56%, 10-y OS 15%	2 pts with G4
Pötter [21]	1999	72	Pure	No	Salivary gland ACC	3-y LC 73%; 5-y OS 58%	4%–6% acute G3–4; 1 temporal lobe necrosis
Douglas [22]	2003	279	Pure	No	Salivary gland ACC	6-y LC 59%; 6-y DFS 67%	10% of late G3–4
Brackrock [23]	2005	71	Pure	No	Salivary gland ACC	6-y LC 45%; 6-y OS 60%	9 late G3–4 events
Timoshchuk [24]	2019	545	Pure	No	Salivary gland ACC	10-y LC 79%; 10-y OS 62%	3%–7% risk of osteradionecrosis
Laramore [25]	1989	38	Mixed	Yes	Brain AA	Median OS 1.7 y	
Breteau [26]	1996	294	Mixed	No	Brain GBM	Median OS 26.7 mo	3 documented radionecrosis
Stelzer [27]	2008	10	Pure	No	Brain GBM	1-y OS 66%	moderate gliosis and microvascular sclerosis at autopsy
Sarycheva [28]	2021	40	Mixed	No	Rec gliomas	3-y OS 66.7%	ND

**Abbreviations**: pts, patients; FNT, fast neutron therapy; H&N, head and neck; c-r, cancer; OS, overall survival; G, grade; LC, local control; ACC, adenoid cystic carcinomas; LRC, local regional control; DFS, disease-free survival; AA, anaplastic astrocytoma; GBM, glioblastoma; Rec, recurrence.

**Table 2.** Studies evaluating fast neutron therapy for various tumor locations.

**Reference**	**Publi cation date**	**N of neutron pts**	**Type of FNT**	**Compared with photons**	**Pathology**	**Outcomes**	**Toxicity**
Russel [29]	1987	45	Mixed	Yes	Prostate c-r	8-y OS 63%	25 events with G3–4
Krieger [30]	1989	55	Mixed	Yes	Prostate c-r	5-y LRC 81%; 5-y OS 70%	9% of G3–4
Laramore [31]	1993	55	Mixed	Yes	Prostate c-r	10-y LC 70%, 10-y OS 46%	8 events of G3, 1 death
Forman [32]	2002	700	Mixed	Yes	Prostate c-r	5-y DFS 93%	0%–18% of G3–4, depending on neutron dose
Caterall [33]	1987	17	Pure	No	LA Breast c-r	Median OS 26 mo	3 pts with skin necrosis, 17 pts with moderate fibrosis
Smirnova [34]	1998	85	Mixed	Yes	LA Breast c-r	5-y OS 71.8; 10-y OS 22.7%	No difference with gamma rays
Murray [35]	2005	12	Pure	Yes	Breast c-r	Median OS 21.5 m	6 pts with G3–4
Ragulin [36]	2015	95	Mixed	Yes	Advanced Breast c-r	10-y OS 32.8%; 10-y DFS 29.5%	No difference with gamma rays
Startseva [37]	2015	108	Mixed	Yes	Breast c-r	10-y OS 70.8%; 10-y LC 98%	No ≥G3
Velikaya [38]	2016	91	Mixed	Yes	LA Breast c-r & rec	Rec—8-y OS 87.6%; LA—7-y OS 85.4%	1 pt with Gr 3
Cohen [39]	1984	51	Pure	No	Sarcomas	LC 44%–50%	9 pts with G3–4, 1 death
Budach [40]	1990	40 Sarcomas; 18 CSA	Mixed	No	Sarcomas, CSA	Sarcomas: LC 69.3%; OS 91%. CSA: LC 55.6%; OS 62.9%	no ≥G3
Steingräber [41]	1996	221	Pure	No	Sarcomas	LC 66%	40% of severe fibrosis
Schwartz [42]	2001	72	Mixed/Pure	No	Sarcomas	4-y OS 66%	15% of G3–4
Morita [43]	1985	45	Mixed	Yes	Uterine c-r	5-y 73%	No difference with gamma rays
Pointon [44]	1985	48	Mixed/Pure	Yes	Bladder c-r	T2 stage: 3-y OS 52%. T3 stage: 3-y OS 43%	10%–37% of G3–4, 4 deaths
Maor [45]	1988	80	Mixed	Yes	LA cervical c-r	2-y LC 45%; median OS 1.9 y	19% of G3–4
Eising [46]	1990	20	Mixed	No	Rectal c-r	Pain-free 9 mo—46%; 1-y OS 56%	2 pts with severe fibrosis
Engenhart [47]	1990	26	Mixed	No	Rec rectal c-r	Pain relief in 22 pts	2 pts with G3–4
Patel [48]	2015	30	Pure	No	Pleural mesothelioma	Median OS 22.1 mo	ND
Choinzonov [49]	2017	45	Mixed	No	Thyroid c-r (postop and rec)	Postop: 5-y OS 70.2%; 5-y LC 52%. rec: 5-y OS 32.8%	81% of skin G2–3
Bittner [50]	2008	20	Pure/mixed with brachytherapy (6 pts)	No	Trachea ACC	5-y OS 89.4%	2 events of late G3–4
Eichhorn [51]	1982	600	Mixed	No	Various	1. Positive effects of mixed RT	
						2. Optimal dosage is 1.2–2.0 Gy	
						3. FNT is better for inoperable pts	
						4. Prophylactic postop RT for radioresistant tumors	
						5. High sensitivity of spinal cord	
						6. Differences in assuming the effects of normal tissue between RT and FNT	
Tsunemoto [52]	1988	1623	Pure	ND	Various	Salivary gland ACC, prostate c-r, sarcomas, melanomas, pancoast tumor—are indications for FNT	
Koh [53]	1994	83 H&N; 99 NSLC; 87 Prostate c-r	Mixed	Yes	H&N, inoperable NSLC, Prostate c-r	H&N: 2-y LC 39%; 2-y OS 36%. NSLC: 2-y LC 85%; 2-y OS 14%. Prostate: 2-y LC 89%; 5-y OS 68%	H&N: 19% of late G3–4. NSLC—ND. Prostate c-r—11% of late G3–4.

**Abbreviations**: pts, patients; FNT, fast neutron therapy; c-r, cancer; OS, overall survival; G, grade; LCR, local regional control; LC, local control; DFS, disease-free survival; LA, locally advanced; rec, recurrence; CSA, chondrosarcoma; ND, no data; postop, postoperative; ACC, adenoid cystic carcinoma; RT, radiation therapy; H&N, head and neck; NSLC, non–small cell lung cancer.

## Fast Neutron Beams, Their Properties, and Radiobiology

Effects of radiation on living tissues depend on multiple factors, including dose levels and microscopic dose distributions, as well as fraction delivery and tissue doubling times. The frequency of lethal events in cells under direct irradiation correlates with LET value. Sparsely and densely ionizing radiation types can be referred to as low-LET and high-LET, respectively. The higher LET, the more severe the DNA damage is and the more complicated the pathways are for its repair. Compared with the conventional low-LET beams, high-LET beams kill cells with higher efficiency [54] explained by indirect oxygen effects and direct damage to DNA [55, 56]. Owing to their direct effect on DNA, high-LET particles act on tumor cells independently of oxygenation, and their relative biological effectiveness (RBE) has little to do with the oxygen enhancement ratio, typically low in the hypoxic tumor microenvironments.

Moreover, high-LET particles have low selectivity regarding cell cycle, affecting cells at different stages of cell cycle progression [57]. This is a consequence of the multiplicity of DNA lesions (clustered DNA damage) produced by single particles. Clinical applications conventionally involve neutrons with RBE = 3 [13], although the value may show complicated behaviors depending on particle energy and fractionation [58–60].

Optimal doses and biological effects of fast neutrons also depend on tissue type; for instance, the dose absorbed in fat is 20% higher than in muscles. Interacting with biomolecules in living tissues, fast neutrons produce protons and the high-LET recoil nuclei of C, N, and O, which poses significant risks of normal tissue side effects [61]. Concurrently, fast neutrons are of historically proven effectiveness toward certain malignancies containing hypoxic cells incredibly resistant to conventional irradiation; the total treatment length can be reduced given the low number of neutron fractions required [62].

Various nuclear reactions (d+T, d+Be, p+Be, and d+T) can be used to obtain fast neutrons in different energy ranges. Production of neutrons by these reactions is accomplished with cyclotrons, linear accelerators, nuclear reactors, or neutron generators. A p+Be reaction is a preferable option for modern, high-energy facilities [63–65].

## Clinical Experience

Shortly after the first cyclotron was developed at the University of California, Berkeley, in 1936, Locher et al [66], Lawrence et al [6], and Robert [67] suggested using both fast and slow neutrons to treat cancer. The first attempts entailed severe complications and the concept stayed frozen for decades. In 1977, Catterall et al [14] described positive outcomes of FNT in a randomized clinical trial comparing the use of fast neutrons versus photons or gamma rays in 102 patients with advanced head and neck (H&N) cancers. The study revealed significant advantages of fast neutrons alongside comparable severity of side effects. The authors promoted FNT as a matter of clinical utility and no longer an experimental approach [14]. However, other clinical results were controversial. Along with the strong long-term response to FNT, a significant proportion of the patients developed severe side effects [68] subsequently linked to several problems (ie, RBE miscalculation, fields overlapping, and high total doses) [58, 59].

Initial estimates of the immediate and delayed RBE of fast neutrons were modeled on photon therapy. The aggravated side effects of FNT have been associated with increased reactivity of normal tissues. In particular, the standard RBE of 3 (conventionally applied to estimate the risks of early and late normal tissue reactions) corresponds to higher de facto effectiveness in the late-responding tissues with a low alpha/beta ratio. At lower doses, the atypically high RBE values also contribute to higher toxicity as compared with photon beams or gamma rays of the same megavoltage. It was eventually suggested to combine fast neutrons with photons or gamma rays, thus reducing the toxicity of FNT to acceptable levels [69, 70].

Throughout the 1980s and 1990s, fast neutron facilities grew in number and FNT became an available option for certain tumors, including advanced prostate cancer, breast cancer, and bone and soft tissue sarcomas. In 1985, Peters et al [69] reviewed FNT studies ongoing in the US. The authors emphasized that the statistically proven benefits of FNT for local tumor control and survival in patients with advanced prostate cancer, H&N tumors, and bladder cancer were dependent on the use of correct treatment schedules and proper neutron sources [69].

Later, Laramore et al [71] summarized the results of FNT for the allegedly radioresistant bone and soft tissue sarcomas. The study involving 652 patients treated with either neutrons or photons showed a 49% to 55% local control (LC) rate in the FNT group versus 21% to 38% in the photon therapy group, depending on the histological type. No significant differences in adverse events were recorded [71]. For brain gliomas, however, FNT was of no benefit; moreover, the neutron boost had a negative impact on survival, directly related to the deteriorating action of neutrons on nervous tissue [25]. Still, given its specific radiobiological features, FNT remained a promising option for other tumors poorly responding to RT. A final report from the Radiation Therapy Oncology Group–Medical Research Council [20] comparing fast neutrons and photons for the treatment of inoperable salivary gland tumors noted a significant advantage of FNT in terms of LC and a marginal benefit in overall survival (OS), ultimately proposing FNT as the treatment of choice for such tumors.

Koh et al [53] published the results of ongoing phase III FNT versus photon trials in squamous cell H&N tumors, non–small cell lung cancers, and prostate adenocarcinomas. Contrary to previous studies [15, 67, 68], FNT did not show any considerable advantage for H&N cancers. Despite its shorter time frame (4 weeks cf. 7 weeks for the photons), the use of FNT was associated with a significant increase in toxicity. Simultaneously, for a subgroup of non–small cell lung cancer patients with inoperable tumors, FNT showed better results in terms of both LC and OS (16% versus 3%, *P* = .02 and 19% versus 6%, *P* = .15, respectively). In addition, there was a trend of better outcomes for neutron-treated patients with unfavorable prognostic factors (eg, T_4_/N_3_ stage, critical condition) [53].

Another seminal paper favoring the use of fast neutrons was published in 1996 [72]. Lindsley et al [72] reported the benefits of FNT in local regional control of unresectable salivary gland tumors compared with photon RT (respectively, 56% versus 17% efficacy). In 1997, Sur et al [73] confirmed the primacy of fast neutrons for salivary gland tumors based on a similar comparative evaluation of FNT and photon RT outcomes.

Several clinical trials of FNT indicated an improvement in OS rates for squamous-cell lung carcinoma [72]. For H&N cancers, fast neutrons showed a statistically significant improvement in the overall response rate as compared with photons (70% versus 52%, respectively), with similar survival rates in both groups [72].

In connection with its minor sensitivity to hypoxia, FNT is of profound interest for the treatment of slow-growing tumors, notably prostate adenocarcinomas [29–31]. Koh et al [53] observed significant advantages of FNT over photons in a cohort of 172 patients with prostate adenocarcinoma (5-year local regional control in 89% versus 68%, respectively), albeit with a higher prevalence of severe complications (11% versus 3% for 5-year observation). In 1998, Lindsley et al [74] published an encouraging summary of worldwide clinical experience of FNT for prostate cancer, notably the benefits of using mixed neutron-photon beams versus pure fast neutron or photon beams.

The striking clinical effects of pure fast neutrons are intrinsically related to the worst issue of FNT—high levels of severe toxicity. Many protocols implemented a combination of conventional photons and fast neutrons, thus reducing the adverse impact of FNT [13].

As the penetrating capacity of neutron beams was initially low, the output energies have eventually increased to dozens of megavolts. The safety issue was relatively settled; paradoxically, it coincided with the beginning of a decline. The new higher mega-electron volt (MeV) neutron units did not provide clear radiobiological benefits in most clinical trials. For instance, Murray et al [35] showed no differences in the use of 66-MeV neutron versus photon irradiation in breast cancer treatment. Besides, the study was underpowered to demonstrate the benefit of FNT over photons, due to premature closure, without reaching its endpoints.

The progressive development of advanced multileaf collimators opened an era of three-dimensional conformal photon irradiation in the beginning of the 2000s [75]. New technical approaches made the conventional RT both deadlier for tumors and safer for the patients. With its evident toxicity and in the absence of explicit guidelines for its use, FNT hardly stayed in focus for anyone except enthusiastically minded experts. With the radiation protection requirements for patients and staff becoming more restrictive, the number of neutron facilities has progressively gone down [76].

Despite the obvious decline, FNT was not completely ruled out. In 2001, Schwartz et al [42] published the outcomes of FNT in 72 patients with soft tissue and cartilaginous sarcomas treated between 1984 and 1996. These tumors show high rates of local aggressiveness and resistance to chemotherapy and RT. As the treatment resulted in a 4-year OS of 66% for the curatively treated group and a 1-year OS of 62% for the palliation group, FNT was recognized as a reliable option for both curative and palliation protocols.

Micke et al [77] presented the outcomes of FNT for local regional recurrences of pre-irradiated H&N cancers with a promising 1-year OS of 29.3% and tolerable side effects. In theory, fast neutrons are most advantageous when applied to hypoxic cell pools that prevail in H&N tumors. On the other hand, a Cochrane Collaboration overview [78] about the efficacy of RT for oral cavity and oropharyngeal cancer failed to find any significant difference in survival or disease-control outcomes between FNT and conventional photon RT.

In 2002, Forman et al [32] published an extensive data set comprising FNT outcomes for 700 prostate cancer patients treated with neutrons only or mixed neutron-photon beams. The authors identified a 50:50 ratio of neutrons and photons (corresponding to 10 Gy of neutrons and 40 Gy of photons) as the optimal tradeoff between tumor control rates and normal tissue adverse events [32]. These were the last attempts at FNT of prostate cancer, which proved an unfavorable option. The effective alternative treatments for prostate cancer available to date (photon external beam RT, brachytherapy) allow high rates of local control without excessive toxicity; hence the lack of need for FNT in this area.

As for other tumor types, the effectiveness of fast neutrons in the adenoid cystic salivary gland cancers was additionally corroborated by Douglas et al [22] and Brackrock et al [23]. An interesting study estimating the outcomes of FNT and brachytherapy for adenoid cystic carcinoma of the trachea was published in 2008 by Bittner et al [50], presenting excellent results, with a 5-year OS of 89.4% and toxicity within grade 3.

It should be emphasized that a majority of studies on FNT published in the 2000s were based on the data collected in the 1980s and 1990s, and only a few of them, enrolling small cohorts of patients, involved neutron units built after 2000. For instance, Stelzer et al [27] applied FNT to treat 10 patients with glioblastoma; the treatment was carried out under positron-emission tomography guidance. Similar to the previous studies on brain tumors, the low-radiation tolerance of brain tissue critically limited the odds of success, even with the modern conformal techniques and positron-emission tomography [27].

In the 2000s, only a few FNT facilities worldwide were still operating—in the US, Germany, and Russia [13]. The clinical experience of FNT in Russia is remarkable due to the extensive nonmilitary nuclear infrastructure historically related to the advanced physical research in the former Soviet Union. Gulidov et al [79] successfully applied fast reactor neutrons from a nuclear power station to different cancers, mostly H&N and breast cancers. Lukina et al [80] presented the results of mixed neutron-photon treatment for 33 patients with soft tissue sarcomas, achieving 100% response and 42% OS. One of the last fast neutron centers was functioning in Tomsk until the 2010s.

Being a relatively superficial RT target, locally advanced breast cancers also showed a good response to FNT. Moreover, breast cancers remain among the tumors most responsive to FNT. For instance, in a study comprising fast reactor neutron and photon arms, enrolling, respectively, 95 and 106 patients with T4N+ breast cancer, the estimated 10-year survival was, respectively, 32.8% versus 17.1% (*P* = .02) [36, 81]. Startseva et al [37] achieved a 70.8% 10-year OS in patients with different stages of breast cancer by using 6.3-MeV FNT as a preoperative modality. For recurrent breast tumors, 8-year OS of 87.6% after FNT was reported by Velikaya et al [38].

However, local therapy generally aims to achieve acceptable local control with minimal adverse impact on a patient's life quality. Similar to the case of prostate cancer, a variety of much safer therapy modalities are now available for breast cancer, and FNT is not on this list. Over the last 2 decades, the only clinical value of FNT has been for rare tumors or cases with critically limited actionable opportunities.

For instance, Liao et al [18] reported 5-year local control after FNT in 66% of patients with H&N mucosal melanoma, albeit at survival rates critically undermined because of early distant progression (5-year OS of 21%). Another fatal tumor, pleural mesothelioma, was treated with neutrons by Patel et al [48]; the outcomes were poor as FNT did not bring survival benefits due to the distant expansion of the tumor.

Macomber et al [82] applied FNT for refractory cutaneous Merkel cell carcinoma in a patient with a previous treatment failure; LC was achieved with 18 Gy in 12 fractions and lasted for 2 years. Schaub et al [83] presented 2 promising observations of Merkel cell carcinoma treated with FNT, noting a potential relationship between high-LET neutron beam irradiation and elevated immune response. Choynzonov et al [49] reported 45 patients with resistant forms of thyroid cancer, 32.8% of them responding to FNT (alone or combined with surgery); the only manifestation of local toxicity was skin epidermitis grade 1 to 3.

Overall, salivary gland tumors show the best rates of local response to FNT (10-year LC of 79%) with improved survival rates (10-year OS of 62%) and osteoradionecrosis incidence comparable to photons (2% versus 7%), based on the data for 545 patients [24]. Until 2015, fast neutrons represented an officially recommended RT option for salivary gland cancers, excluded from the guidelines mainly due to its low availability [84].

## Prospects

In the new millennium, hadron therapy is a constant focus of attention [85, 86]. The latest technical improvements, cost reduction for the facilities development, and the increasing clinical evidence make the unconventional irradiation quite promising. Alongside protons, heavy carbon ions, and epithermal neutrons, fast neutrons appear highly relevant as well, especially for extremely aggressive and therapy-resistant tumors.

One of the clues to better understanding the clinical controversy associated with FNT lies in the methods of beam generation. The available sources of fast neutrons include cyclotrons, linear proton accelerators, nuclear station reactors, and specific neutron generators. The biological effects of high-LET beams strongly depend on their source, energy, and fractionation. The differences in beam characteristics are partially responsible for the controversy of clinical evidence.

Owing to the inverse dose per fraction effect on RBE, especially in late-responding normal tissues, the trend has eventually shifted toward the use of smaller doses per fraction, with the optimal 10 to 18 Gy corresponding to 1 to 1.8 Gy per fraction [87]. The small-dose schemes of FNT allowed standardization of the treatment and reduced severity of side effects. Mixed irradiation with neutrons and photons or gamma rays, featured in several clinical trials, proved as effective as pure neutrons while being remarkably less toxic as compared with the early low-energy neutron units with inferior beam characteristics and primitive delivery equipment, which negatively affected the clinical outcomes [62, 88].

As fast neutrons have been invariably associated with severe early and late toxicity, it would be reasonable to expect the concomitantly increased risks of radiation-related secondary tumors. Indeed, some of the long-term analyses of secondary malignancies in the aftermath of FNT showed such an increase. For instance, MacDougall et al [89] studied the delayed effects of FNT in 620 patients after RT, with a significant increase of secondary tumors in the FNT population. Similarly, Expósito et al [90] reported an increase in the rates of secondary malignancies based on follow-up clinical data for 1377 patients after FNT.

Despite the allegation of high toxicity of FNT [91, 92] (based on largely outdated sets of clinical evidence in terms of irradiation regimens and equipment used for neutron beam generation), FNT holds great promise for the treatment of various radioresistant tumors with critically limited therapeutic options [93]. Contemporary studies continue to accumulate essential radiobiological data on high-LET therapy involving proton and C-ion irradiation applicable to FNT of similar parameters (eg, Bragg peak, ionization density) [94, 95].

Fast neutron beam radiobiology has multiple abovementioned uncertainties, which are challenging to address. The last decades brought such innovations as advanced multileaf collimators, new means for the neutron beam intensity modulation, and new toxicity correction methods [75, 96–98]. The recent introduction of the Monte-Carlo–based neutron treatment planning system will potentially facilitate the FNT reinvention [93, 99]. Most of the previous FNT results were achieved without even seeing the isodose levels. Recent advances in the intensity modulation allow a 20% to 80% reduction of the fast-neutron dose to susceptible organs at risk in the H&N area [100]. The latest technical solutions can make neutron generators compact and conveniently transformed into portable cost-effective clinical units [101, 102].

## Conclusion

Although fast neutrons have a bad name due to their controversial clinical track record, their overall potential in cancer treatment seems to be underestimated. Based on radiobiological data, fast neutrons might kill tumor cells with comparable effectiveness as the C-ions. Fast neutrons, an established modality for the treatment of certain tumors, can be used for rare radioresistant superficially located tumors, without obvious effective treatment or as a boost in combination with less toxic irradiation modalities. With technological upgrades and randomized trials, fast neutrons are guaranteed a limited, but special, place in modern radiation oncology.
